# Structural impact of non-IID heterogeneity on federated behavioral anomaly detection in IoT and IoMT systems

**DOI:** 10.3389/frai.2026.1825067

**Published:** 2026-06-18

**Authors:** Jorge Robalino-Díaz, Alejandro Cabrera-Andrade, Sergio Luján-Mora, William Villegas-Ch

**Affiliations:** 1 Escuela de Ingeniería en Ciberseguridad, FICA, Universidad de Las Américas, Quito, Ecuador; 2 Carrera de Enfermería, Facultad de Ciencias de la Salud, Universidad de las Américas, Quito, Ecuador; 3 Grupo de Bio-Quimioinformática, Universidad de las Américas, Quito, Ecuador; 4 Departamento de Lenguajes y Sistemas Informáticos, Universidad de Alicante, Alicante, Spain

**Keywords:** artificial intelligence, behavioral anomaly detection, explainable artificial intelligence (XAI), federated learning, IoMT security

## Abstract

The expansion of Internet of Things (IoT) and Internet of Medical Things (IoMT) infrastructures has increased the generation of multivariate sensor streams that reflect complex operational behaviors in industrial and clinical environments. Centralized anomaly detection approaches face limitations in IoMT due to privacy constraints, latency, and device heterogeneity. Federated learning (FL) enables distributed model training without data centralization; however, its behavior under highly non-Independent and Identically Distributed (non-IID) conditions remains insufficiently understood. This study proposes a trace-level behavioral modeling approach combined with federated training via FedAvg to analyze the impact of non-IID heterogeneity on anomaly detection. An Integrated Hybrid Dataset (IHD) comprising 71,980 behavioral traces, with 22,698 used for evaluation, was constructed from Edge-IIoTset, TON_IoT, and IoMT data. The centralized model achieved *F*_1_ = 0.981 and Recall = 0.993, while the federated model preserved discriminative capacity (AUC-ROC = 0.995) but reduced Recall to 0.530. Degradation is concentrated in IoMT (Recall = 0.290), with increased Brier Score and Expected Calibration Error, showing that preserved discrimination does not ensure operational effectiveness.

## 1 Introduction

The sustained expansion of Internet of Things (IoT) infrastructures, and particularly the Internet of Medical Things (IoMT), has transformed cyber-physical systems into highly instrumented environments where large-scale streams of sensor events capture real-time operational dynamics. In industrial and clinical domains, these multivariate flows not only reflect physical states but also encode behavioral patterns that evolve under varying conditions of load, context, and risk (Yacoubi et al., 2026a). In such environments, security increasingly requires modeling normal behavior and identifying structural deviations, rather than relying exclusively on static inspection or signature-based detection.

Traditional centralized architectures have historically dominated anomaly detection in IoT systems. However, their applicability in IoMT is constrained by the centralization of sensitive clinical data, latency limitations, and scalability challenges under heterogeneous device distributions. Recent studies highlight that privacy, trust, and regulatory compliance constitute fundamental barriers to the adoption of centralized approaches in medical environments (Jiang et al., 2026; Shammar et al., 2025). These constraints motivate the adoption of distributed learning paradigms that preserve local data while enabling collaborative model construction.

Federated learning (FL) enables distributed training without exchanging raw data (Sabah et al., 2025). Nevertheless, standard aggregation schemes such as FedAvg are known to be sensitive to non-Independent and Identically Distributed (non-IID) data, particularly when class prevalence differs significantly across clients (Chowdhury et al., 2026; Alzakari et al., 2025). In IoMT environments, such heterogeneity is structural, as certain clients may exhibit extreme dominance of anomalous or benign behaviors. Despite this, most studies emphasize convergence or loss minimization, without systematically analyzing how non-IID conditions affect operational performance, calibration, and domain-level sensitivity.

A key problem in this context is therefore not limited to the applicability of FL in IoT-IoMT environments, but to the characterization of its operational behavior under heterogeneous conditions (Zukaib et al., 2024). In particular, it is necessary to determine whether federated models can preserve discriminative capacity while maintaining sensitivity to anomalies across domains with extreme prevalence asymmetries, and whether degradation is uniformly distributed or concentrated in specific clients due to weighted aggregation effects.

To address this problem, this work proposes a distributed analytical framework that integrates trace-level behavioral modeling and federated training using FedAvg. The objective is not to optimize performance, but to systematically characterize the interaction between non-IID heterogeneity, probabilistic calibration, and operational sensitivity in IoT and IoMT environments. For this purpose, an Integrated Hybrid Dataset (IHD) was constructed by combining processed subsets of Edge-IIoTset, TON_IoT, and IoMT data, resulting in 71,980 behavioral traces, from which 22,698 are used for evaluation.

The empirical evaluation reveals a divergence between statistical discrimination and operational performance. While the centralized model achieves *F*_1_ = 0.981 and Recall = 0.993, the federated model maintains a high AUC-ROC = 0.995, indicating preserved ranking capability, but exhibits a substantial reduction in Recall to 0.530 (*F*_1_ = 0.692). Domain-level analysis shows that IoT maintains near-centralized performance (*F*_1_ = 0.955), whereas IoMT experiences a pronounced reduction in sensitivity (Recall = 0.290), indicating a shift in decision behavior under a fixed threshold.

Additional analysis of training dynamics shows that the federated model traverses intermediate high-performance states before reaching a configuration with reduced sensitivity in IoMT. Calibration metrics further indicate that this degradation extends beyond classification performance, with increases in Brier Score and Expected Calibration Error (ECE), reflecting instability in probabilistic outputs under non-IID conditions. Additional feature-level attribution analysis reveals that federated aggregation also redistributes the relative contribution hierarchy associated with anomaly discrimination, amplifying selected network-level descriptors while altering the contribution balance between physiological and behavioral features in the IoMT domain. These results highlight that preserved discriminative capacity does not guarantee operational effectiveness, and that both probabilistic calibration and feature-level contribution redistribution play a central role in characterizing performance degradation under FL.

The work is organized as follows. Section 2 reviews related work on FL and anomaly detection in IoT and IoMT environments. Section 3 describes the materials and methods. Section 4 presents the experimental results. Section 5 discusses the findings. Section 6 concludes the paper.

## 2 Literature review

Security in IoT and Industrial Internet of Things (IIoT) environments has increasingly been reframed as a big-data analytics problem, driven by the heterogeneity of devices, the continuous nature of data flows, and the need for near-real-time responses. Recent studies propose a distributed architecture based on edge and fog computing, in which deep learning and temporal analysis techniques enable the processing of large volumes of data without relying solely on centralized infrastructure (Gokila et al., 2024). In this sense, the notion of “cognition” is often associated with the system's ability to adapt dynamically to changes in observed behavior.

However, a recent systematic review shows that, in practice, these approaches continue to prioritize classic performance metrics, such as accuracy or F1-score, as the main criterion for success, relegating the interpretation of anomalous behavior to a secondary role (Ogunseyi et al., 2026). Even when attention or sequential analysis mechanisms are introduced, behavior is mostly represented as a classification instance, without explicit modeling of its evolution or its operational significance in distributed scenarios. As a result, current approaches remain focused on optimizing the detection model, without formalizing a process for generating and reusing security knowledge across distributed environments.

This emphasis on algorithmic performance reveals a conceptual gap: literature acknowledges the cognitive complexity of IoT/IIoT environments but rarely translates this into mechanisms that enable reasoning about anomalies as persistent, shareable behavioral patterns across nodes (Doménech et al., 2024; Boubertakh et al., 2026). Meanwhile, FL has established itself as a central strategy for anomaly detection in IoT and IoMT, primarily due to privacy, regulatory, and data-sovereignty constraints. Recent work explicitly highlights that the data generated in these environments is inherently non-IID due to the diversity of devices, operating contexts, and usage profiles (Misbah et al., 2025). Based on this premise, federated architectures are proposed that enable training global models without centralizing sensitive data.

Despite these advances, a critical analysis of the literature shows that the treatment of non-IID data is predominantly approached from a statistical perspective, with a focus on preserving training stability and overall model performance (Farooqi et al., 2025). Mechanisms such as secure aggregation, differential privacy, and blockchain aim to ensure trust and robustness, but they do not challenge the implicit homogenization introduced by federated consensus (Alsharidah et al., 2025; Goel and Neduncheliyan, 2025).

Most proposals assume that anomalies detected across different nodes are semantically comparable, whereas their meaning can depend heavily on the local domain or specific operational state (Doménech et al., 2024; Boubertakh et al., 2026). This limitation is especially critical in industrial and medical settings, where rare or localized anomalies can have disproportionate consequences. Thus, current FL tends to optimize models rather than preserve the behavioral diversity necessary for informed and distributed decision-making.

Furthermore, the incorporation of explainable artificial intelligence techniques into distributed security systems has emerged as a response to the opacity of deep learning models, especially in critical environments. Recent studies have integrated Post-hoc techniques such as SHAP and LIME into federated architectures to increase transparency and facilitate decision auditing (Ogunseyi et al., 2026; Yacoubi et al., 2026b). However, analysis of these studies reveals that explainability is typically treated as an afterthought. Explanations are generated locally to justify individual predictions, but they are neither integrated into the federated process nor used as input for aggregating, validating, or adapting the global model (Farooqi et al., 2025). Furthermore, recent reviews highlight the lack of standardized metrics for evaluating the quality and usefulness of explanations in real-world operational scenarios, thereby limiting their practical impact (Ogunseyi et al., 2026; Duan et al., 2023).

This fragmentation reduces the cognitive potential of XAI, which remains disconnected from the learning cycle and decision-making. Instead of contributing to distributed reasoning, explanation is limited to justifying model outputs, without facilitating a shared understanding of anomalous behavior or supporting coordinated responses. In the IoMT domain, recent literature emphasizes that safety must be addressed as a sociotechnical problem, in which anomaly detection is directly linked to patient safety and the continuity of clinical processes (Misbah et al., 2025). Behavior-based approaches are recognized as promising, but they face structural limitations, such as the scarcity of open datasets specific to IoMT and the high heterogeneity of devices and contexts (Goel and Neduncheliyan, 2025). Within this landscape, the interaction between non-IID heterogeneity, probabilistic calibration, and domain-level sensitivity in federated IoT/IoMT environments remains insufficiently characterized, particularly under extreme prevalence conditions (Yacoubi et al., 2026b; Cunha et al., 2025).

The reviewed literature reveals a consistent focus on optimizing global performance metrics and ensuring training stability in federated IoT and IoMT environments, while leaving several critical aspects insufficiently explored. In particular, limited attention has been given to how non-IID heterogeneity affects the relationship between discriminative capacity, probabilistic calibration, and domain-level sensitivity, especially in high-risk scenarios such as IoMT. Furthermore, existing approaches rarely examine how these factors interact dynamically during federated training or how they impact operational reliability across heterogeneous clients. In response to these gaps, this study provides a structured analysis of federated anomaly detection that explicitly investigates the interaction between non-IID data distributions, calibration behavior, and domain-specific performance, offering a more comprehensive characterization of model behavior in heterogeneous IoT and IoMT environments.

## 3 Materials and methods

### 3.1 Conceptual framework and research design

This work adopts a computational-methodological design in which security in IoT and IoMT environments is modeled as a distributed analytical pipeline that integrates behavioral modeling, FL, and probabilistic analysis. Rather than framing anomaly detection as an isolated classification task, the proposed design focuses on how detection performance, calibration, and domain sensitivity evolve under heterogeneous and non-IID conditions. This perspective is motivated by recent findings in IoMT security, where structural challenges extend beyond predictive accuracy to include traceability, reliability, and privacy preservation in distributed clinical environments (Shammar et al., 2025). In parallel, prior work on FL in IoT has shown that the interaction between heterogeneity and aggregation strategies can significantly affect operational stability if not explicitly characterized (Alatawi, 2025a).

**Figure 1:**
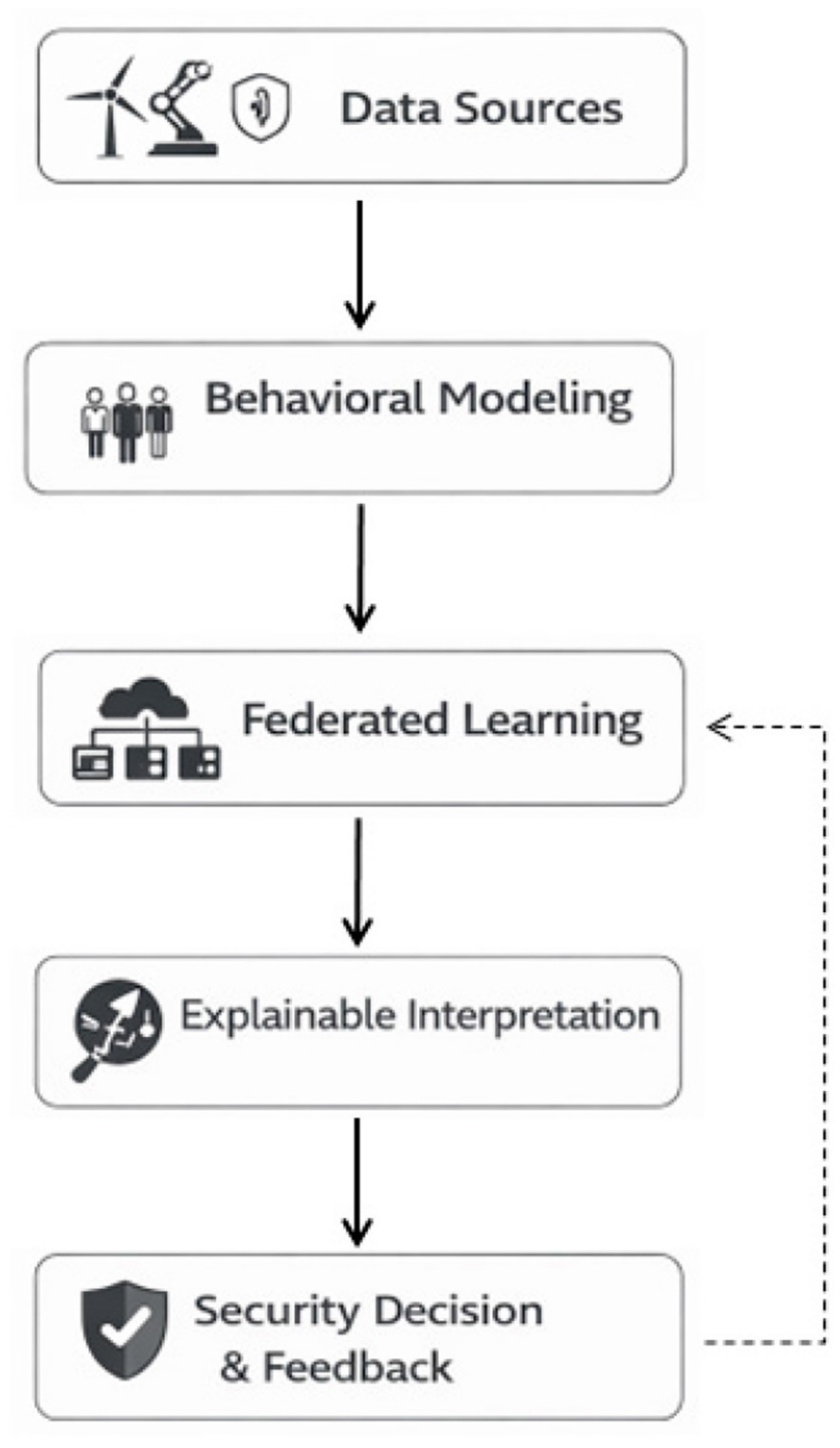
Distributed analytical process for security modeling in IoT and IoMT environments.

Within this framework, security is treated as a transformation process in which raw data is converted into structured behavioral representations that support distributed learning and evaluation. As illustrated in Figure 1, the process begins with heterogeneous data sources from IoT and IoMT devices that differ in sampling frequency, communication protocols, and operational contexts. These data streams are transformed into temporal behavioral traces through fixed-length windowing, in which each trace aggregates statistical features such as event counts, durations, and activity patterns over a defined interval, enabling the representation of system dynamics and facilitating the identification of deviations over time.

Behavioral modeling constitutes the first stage of the pipeline. At this stage, low-level measurements are aggregated into fixed-length temporal windows, producing trace-level representations that capture device activity over time. This transformation enables anomaly detection on sequences rather than isolated observations, which is better suited to capturing structural deviations in dynamic environments.

Given the distributed nature of IoT and IoMT systems and the sensitivity of the data involved, FL is employed as the training mechanism. In this study, FL is implemented using the standard FedAvg algorithm, in which local models are trained independently on each client using their corresponding behavioral traces and aggregated through weighted averaging. This baseline configuration allows isolating the impact of data heterogeneity on model behavior, without introducing additional optimization mechanisms that could mask the effects of non-IID distributions.

The dataset is partitioned at the client level, and train/test splits are performed independently for each client using stratified sampling. All classification metrics are computed using a fixed decision threshold of 0.5 applied to predicted probabilities.

To support the interpretation of model behavior across domains, the framework incorporates a structural analysis approach based on probabilistic outputs, calibration metrics, and score distributions. This approach primarily focuses on analyzing prediction distributions and calibration behavior across domains and training schemes, complemented by feature-level attribution to characterize shifts in internal contributions under federated aggregation.

The final stage of the pipeline involves evaluating detection performance and its evolution during federated training. Metrics such as F1-score, Recall, and AUC-ROC are used to characterize discriminative capacity, while Brier Score and ECE are used to assess probabilistic reliability. In addition, round-by-round analysis is conducted to examine convergence dynamics and parameter stability under non-IID data distributions. This design enables a systematic characterization of FL behavior in heterogeneous IoT and IoMT environments.

### 3.2 Data sources and behavioral modeling

#### 3.2.1 Data sources and integrated hybrid dataset

The proposed analysis is based on publicly available datasets widely used in IoT and IoMT security research, selected for their ability to represent heterogeneous and distributed operational conditions. Edge-IIoTset and TON_IoT (Ferrag et al., 2022; Moustafa, 2019) are used to represent IoT/IIoT environments. In contrast, the IoMT domain is represented by a derived dataset constructed from medical-device traffic logs and patient monitoring alerts, referred to as the Patients+Alerts dataset within the IHD pipeline. This dataset does not correspond to a standardized public benchmark, but rather to a locally curated collection of clinical device communication records and alert events, designed to reflect realistic IoMT operating conditions. It includes device-level interaction patterns, alert signals, and temporal activity records commonly observed in patient monitoring systems.

Edge-IIoTset provides multilevel data at the network, system, and application layers, including both normal and anomalous scenarios, enabling the analysis of interaction patterns and temporal behavior in IoT environments. TON_IoT complements this by integrating multimodal sources such as network traffic, telemetry, and system logs, allowing cross-layer behavioral correlations. The IoMT component captures operational patterns specific to clinical environments, where deviations may reflect both security incidents and abnormal device behavior.

The IHD is constructed through a structured data integration process. Rather than merging raw records, the datasets are harmonized at the representation level through schema alignment, feature selection, and normalization. Each record retains provenance metadata, including domain (IoT or IoMT), device type, and federated client identifier, ensuring traceability and enabling distributed partitioning.

To ensure compatibility across datasets, features are standardized through *z*-score normalization using global statistics computed from the training data. Continuous variables are scaled using mean and standard deviation, while categorical variables are encoded using consistent mapping schemes across domains. Labels are unified into a binary anomaly-detection setting, where anomalous and normal classes are consistently defined across all sources. This process results in a unified dataset comprising 71,980 behavioral traces, of which 22,698 are used for evaluation.

#### 3.2.2 Behavioral representation

Behavioral modeling is formulated by representing IoT and IoMT activity as sequences of events rather than isolated observations, enabling the capture of dynamic behavioral patterns. Each observation is defined as a multidimensional vector (Equation 1):


$$\begin{array}{l} x_{t}\in {\mathbb{R}}^{d} \end{array}$$ (1)

where *d* denotes the number of observable attributes associated with an event index *t*.

To incorporate sequential structure, events are grouped into fixed-length windows defined over event counts, as shown in Equation 2:


$$\begin{array}{l} W_{i}=\left\{x_{t}|t\in \left[t_{i},t_{i}+\Delta \right]\right\} \end{array}$$ (2)

where Δ denotes the number of consecutive events included in each window. In this study, Δ = 500 events are used to construct behavioral traces. This choice ensures that each trace captures sufficient activity to represent device behavior while maintaining a consistent aggregation granularity across heterogeneous datasets.

Each window is then transformed into a behavioral trace through an aggregation function, as defined in Equation 3:


$$\begin{array}{l} B_{i}=\phi \left(W_{i}\right) \end{array}$$ (3)

where ϕ(·) produces a fixed-dimensional representation by combining statistical descriptors of the events within the window. These descriptors include mean, standard deviation, minimum, maximum, median, event frequency, and ratio-based measures capturing the proportion of anomalous events within the trace. This aggregation compresses heterogeneous event streams into compact representations while preserving discriminative behavioral patterns.

To enable joint analysis across domains, all behavioral traces are mapped into a unified feature space, as defined in Equation 4:


$$\begin{array}{l} B_{i}^{\left(k\right)}\in {\mathbb{R}}^{m},\;k\in \left\{\text{IoT},\text{IoMT}\right\} \end{array}$$ (4)

where *m* denotes the dimensionality of the transformed representation after feature harmonization across datasets. This unified space ensures compatibility across domains while preserving domain-specific statistical characteristics.

The dataset is partitioned into client-specific subsets, as defined in Equation 5:


$$\begin{array}{l} D_{c}=\left\{B_{i}|\text{source}\left(B_{i}\right)=c\right\} \end{array}$$ (5)

where each client *c* corresponds to a device, domain, or logical subset of the data, this partition preserves the non-IID structure of the dataset, ensuring that each client exhibits a distinct local distribution that directly influences the federated learning process.

### 3.3 Federated learning-based anomaly detection architecture

**Figure 2:**
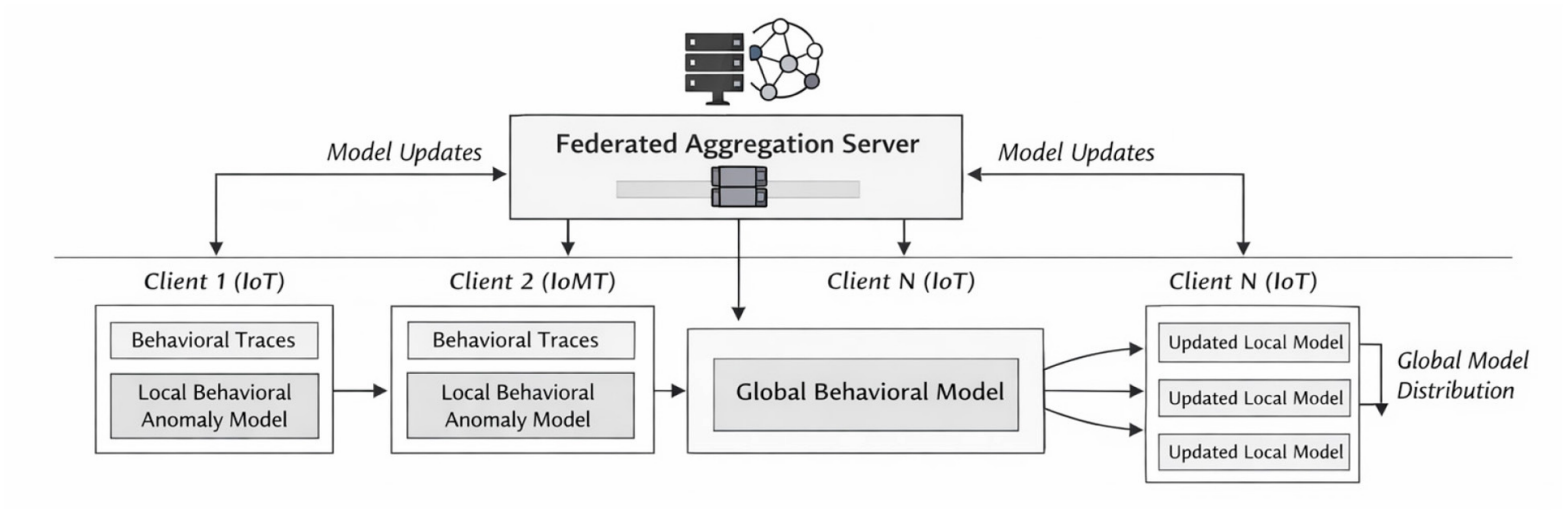
Federated learning-based architecture for behavioral anomaly detection in IoT and IoMT environments.

The proposed architecture employs a horizontal FL scheme that operates on predefined behavioral representations, in which distributed clients collaborate to train a global anomaly detection model without sharing raw data. As illustrated in Figure 2, the system comprises multiple clients distributed across heterogeneous IoT and IoMT domains, along with a central aggregation node that coordinates training. Each client *c* maintains a local dataset of behavioral traces $D_{c}$, preserving both data privacy and the statistical characteristics of its operating environment, which is essential under the heterogeneous and non-IID conditions.

Anomaly detection is formulated as a probabilistic binary classification task defined over behavioral traces. Given an input trace $B_{i}\in {\mathbb{R}}^{m}$, the model produces a probabilistic output ŷ_*i*_∈[0, 1], representing the likelihood of anomalous behavior. The predictive function is defined as $f_{\theta }:{\mathbb{R}}^{m}\to \left[0,1\right]$, parameterized by θ, which is instantiated as a logistic regression model. All classification decisions are obtained by applying a fixed decision threshold of 0.5 to the predicted probabilities.

The anomaly detection model is implemented as a logistic regression classifier operating on the behavioral trace representation. Given an input $B_{i}\in {\mathbb{R}}^{m}$, the model estimates the probability of anomalous behavior as defined in Equation 6:


$$\begin{array}{l} {ŷ}_{i}=\sigma \left(w^{T}B_{i}+b\right) \end{array}$$ (6)

where *w*∈ℝ^*m*^ and *b*∈ℝ denote the model parameters, and σ(·) is the sigmoid activation function. The model is trained using binary cross-entropy loss, as defined in Equation 8, and optimized independently at each client during local updates. Standard logistic regression settings with L2 regularization are used, without additional tuning to preserve consistency across centralized and federated configurations.

For each federated round *r*, local model training is independently performed at each client *c* by minimizing a binary cross-entropy objective over its local dataset, as defined in Equation 7:


$$\begin{array}{l} \theta_{c}^{\left(r\right)}=\arg\underset{\theta }{\min}L_{c}\left(\theta |D_{c}\right) \end{array}$$ (7)

where the local loss function is defined as follows (Equation 8):


$$\begin{array}{l} L_{c}\left(\theta \right)=-\frac{1}{\left|D_{c}\right|}\underset{i\in D_{c}}{\sum }\left[y_{i}\log\left({ŷ}_{i}\right)+\left(1-y_{i}\right)\log\left(1-{ŷ}_{i}\right)\right] \end{array}$$ (8)

This formulation allows each client to learn behavioral patterns that are specific to its local data distribution, without assuming homogeneity across clients. Given the strong non-IID characteristics of IoT and IoMT environments, these local updates inherently capture domain-specific patterns and statistical variations.

Global aggregation is performed using the standard FedAvg strategy, in which local models are combined through a weighted average proportional to the size of each client's dataset, as defined in Equation 9:


$$\begin{array}{l} \theta^{\left(r+1\right)}=\overset{C}{\underset{c=1}{\sum }}\frac{\left|D_{c}\right|}{\overset{C}{\underset{j=1}{\sum }}|D_{j}|}\theta_{c}^{\left(r\right)} \end{array}$$ (9)

This aggregation mechanism ensures that clients with larger datasets have a proportionally greater influence on the global model, providing a controlled baseline for analyzing the effects of non-IID heterogeneity without introducing additional regularization or personalization mechanisms that could obscure these effects.

Once the global model θ^(*r*+1)^ is obtained, it is redistributed to all clients, as defined in Equation 10:


$$\begin{array}{l} \theta_{c}^{\left(r+1\right)}\leftarrow \theta^{\left(r+1\right)} \end{array}$$ (10)

allowing each node to update its local parameters and continue training. This iterative cycle of local optimization, global aggregation, and redistribution defines the FL process evaluated in this study.

Within this architecture, anomaly detection is treated as a probabilistic inference problem over behavioral traces, where anomalies correspond to deviations from learned patterns in the feature space. This formulation enables consistent evaluation across domains while preserving local variability and provides a controlled setting for analyzing how FL behaves under heterogeneous IoT and IoMT conditions, particularly with respect to discriminative capacity and probabilistic calibration.

### 3.4 Structural analysis and interpretation of model behavior

To support the analysis of model performance in heterogeneous IoT and IoMT environments, this work incorporates a structural framework based on probabilistic outputs, calibration metrics, and score distributions. The primary interpretation framework is based on probabilistic outputs, calibration metrics, and score distributions, complemented by a feature-level attribution analysis to characterize changes in the internal contribution structure under federated aggregation.

Given a behavioral trace *B*_*i*_, the trained model produces a probabilistic output, as defined in Equation 11:


$$\begin{array}{l} {ŷ}_{i}=f_{\theta }\left(B_{i}\right) \end{array}$$ (11)

where ŷ_*i*_∈[0, 1] represents the estimated likelihood of anomalous behavior. These probabilities are used not only for classification, but also to analyze calibration and decision consistency under a fixed decision threshold of 0.5.

Calibration is evaluated using the Brier Score and ECE, which quantify the alignment between predicted probabilities and observed outcomes. The ECE is computed by partitioning predictions into bins and evaluating the difference between predicted confidence and empirical accuracy within each bin, enabling the identification of overconfident or underconfident predictions across domains.

In addition to calibration metrics, the distribution of predicted scores is analyzed separately for anomalous and normal traces. This analysis enables the identification of structural shifts in the distribution of predicted probabilities, particularly in federated settings where non-IID data affects the probabilistic output space. For instance, a concentration of anomaly scores below the decision threshold indicates that the model preserves ranking capability (as reflected by AUC) while reducing classification sensitivity (as reflected by Recall).

To further characterize the learning process, the evolution of model parameters is tracked across federated rounds. The change in model weights is quantified using the Euclidean distance between consecutive parameter vectors, as defined in Equation 12:


$$\begin{array}{l} \Delta W^{\left(r\right)}=||\theta^{\left(r\right)}-\theta^{\left(r-1\right)}|{|}_{2} \end{array}$$ (12)

This measure provides insight into convergence behavior and enables the identification of instability patterns associated with heterogeneous data distributions.

Finally, domain-specific analysis is conducted by evaluating performance and calibration separately for IoT and IoMT subsets. This allows a detailed examination of how FL affects each domain differently, particularly under conditions of extreme class imbalance and structural heterogeneity.

### 3.5 SHAP-based feature attribution analysis

Feature-level attribution analysis was performed to characterize the internal contribution structure underlying behavioral anomaly discrimination in centralized and federated learning. Given that the anomaly detector is a logistic regression classifier operating on standardized behavioral trace descriptors, exact SHAP values can be computed analytically, enabling direct estimation of the contribution of each feature to the prediction.

Let $x_{i}\in {\mathbb{R}}^{d}$ denote the standardized behavioral representation associated with trace *i*, and let β_*j*_ represent the coefficient associated with feature *j*. The contribution of feature *j* to the prediction associated with trace *i* is estimated as shown in Equation 13:


$$\begin{array}{l} \phi_{j}\left(x_{i}\right)=\beta_{j}\left(x_{ij}-E\left[x_{j}\right]\right) \end{array}$$ (13)

where ϕ_*j*_(*x*_*i*_) denotes the SHAP attribution associated with feature *j*, and *E*[*x*_*j*_] corresponds to the expected value of the standardized feature computed over the training distribution. Since the behavioral descriptors are standardized using global statistics derived from the federated training partition, the expected feature value remains approximately centered at zero, allowing the attribution structure to be directly interpreted as the interaction between feature magnitude and the learned model coefficients.

The attribution analysis was conducted on anomalous IoMT traces (*y* = 1) extracted from the test partition to characterize the contribution behavior of the domain exhibiting the highest calibration instability and recall degradation. For each training scheme, the mean absolute SHAP contribution was computed across all anomalous IoMT traces to estimate the global magnitude of the contribution associated with each behavioral descriptor.

To quantify the variation in feature contribution induced by federated aggregation, the attribution shift associated with feature *j* was defined as shown in Equation 14:


$$\begin{array}{l} \Delta \phi_{j}=|\phi_{j}^{\left(\text{fed}\right)}|-|\phi_{j}^{\left(\text{cent}\right)}| \end{array}$$ (14)

where $\left|\phi_{j}^{\left(\text{fed}\right)}\right|$ and $\left|\phi_{j}^{\left(\text{cent}\right)}\right|$ denote the mean absolute SHAP contribution of feature *j* under federated and centralized training, respectively. Higher values of Δϕ_*j*_ indicate increased feature contribution under federated aggregation, whereas lower values indicate relative contribution stability across training schemes. In addition to attribution magnitude, the relative ranking position of the dominant behavioral descriptors was analyzed to characterize structural changes in the contribution hierarchy associated with non-IID federated learning.

### 3.6 Experimental setup and evaluation protocol

The evaluation of the proposed approach is designed to assess anomaly-detection performance and model behavior under centralized and federated training schemes. The experimental protocol is structured to isolate the impact of the learning paradigm while maintaining consistency in data representation, model configuration, and evaluation criteria across all settings.

Two experimental scenarios are considered. In the centralized scenario, the IHD is used as a unified dataset for training and evaluation. Behavioral traces are processed through a single learning process without partitioning, allowing the model to operate under conditions of full data availability and providing a reference for performance under ideal data access.

In the federated scenario, the IHD is partitioned into local datasets $D_{c}$, each associated with a different client and representing heterogeneous sources across the IoT and IoMT domains. Each client performs local training using its own behavioral traces, and only model parameters are shared with a central aggregation node. The global model is updated iteratively via weighted averaging of local models, preserving the data's non-IID characteristics. In both scenarios, the same behavioral representation, model architecture, and loss function are used, ensuring that performance differences are attributable solely to the learning scheme.

The dataset is divided into training and test subsets using a stratified splitting strategy applied independently to each client. A fixed random seed and a 20% test proportion are used to ensure reproducibility and preservation of local class distributions. In cases where a client contains only one class, a randomized split is applied to maintain a consistent evaluation protocol.

To ensure comparability between centralized and federated settings, feature standardization is performed using statistics computed exclusively from the training data. These statistics are then applied uniformly across all training and test sets, preventing information leakage while maintaining consistent feature scaling.

Training in the centralized scenario is performed via iterative optimization over the entire dataset. In the federated scenario, training follows an iterative process consisting of local updates and global aggregation across multiple communication rounds. In each round, clients perform local optimization using the same model and loss function, followed by aggregation of model parameters at the central node.

In the federated training process, all clients participate in every communication round (full participation setting), ensuring that all client distributions are represented in each aggregation step. Client selection is therefore deterministic and fixed across rounds. Each client performs a fixed number of local training epochs per round using the same optimization configuration, without adaptive learning rate schedules or dynamic training adjustments. This controlled setting allows the observed variations in performance, including non-monotonic convergence patterns, to be interpreted as resulting from the interaction between non-IID data distributions and the aggregation process, rather than stochastic variations in training dynamics.

Model evaluation is performed on the test set using a fixed decision threshold of 0.5 applied to predicted anomaly probabilities. Detection performance is measured at the trace level using Recall, F1-score, and AUC-ROC. In addition, calibration metrics such as Brier Score and ECE are used to assess the reliability of predicted probabilities.

Beyond aggregate metrics, the evaluation includes domain-specific analysis by separating IoT and IoMT subsets. This enables examination of how model performance varies across domains with different data distributions. Additionally, the evolution of model behavior across federated rounds is analyzed to characterize convergence dynamics and identify instability patterns associated with non-IID data.

## 4 Results

### 4.1 Experimental scenarios and data distribution analysis

The experimental setup yielded a federated topology comprising 10 logical clients (C1–C10) distributed across heterogeneous operating domains. Clients C1–C4 correspond to Edge-IIoT traffic partitions, C5–C8 represent TON_IoT (IoT, Network, Linux, Windows) subdomains, and C9–C10 correspond to patient-centric logs from the IoMT domain. The resulting dataset contains 1,010,000 IoT events and 50,000 IoMT records, producing a total of 1,060,000 events before trace-level aggregation.

Table 1 presents the client-specific distribution of events and behavioral traces under the federated scheme. The volume imbalance is immediately apparent. Client C6 handles 460,000 events, making it the largest single partition. C5 and C7 follow, with 140,000 and 120,000 events, respectively. In contrast, IoMT clients (C9 and C10) record 24,793 and 25,207 events, each representing less than 3% of the total event volume. These distributions correspond to the pre-aggregation event-level structure used to construct the trace-level IHD employed in subsequent experiments.

**Table 1:** Structural distribution of events and behavioral traces across federated clients (IoT and IoMT domains).

Client	Domain	Events (*n*)	Behavioral traces (*n*)
C1	IoT (Edge-IIoTset)	56,500	113
C2	IoT (Edge-IIoTset)	60,500	121
C3	IoT (Edge-IIoTset)	63,000	126
C4	IoT (Edge-IIoTset)	70,000	140
C5	IoT (TON_IoT – IoT)	140,000	280
C6	IoT (TON_IoT – Network)	460,000	20,880
C7	IoT (TON_IoT – Linux)	120,000	240
C8	IoT (TON_IoT – Windows)	40,000	80
C9	IoMT (Patients + Alerts)	24,793	24,793
C10	IoMT (Patients + Alerts)	25,207	25,207

Cumulative concentration reinforces this asymmetry. The three largest clients (C6, C5, and C7) account for 720,000 events, representing approximately 67% of the global total. The four Edge-IIoT partitions combined (C1–C4) record approximately 250,000 events, while the entire IoMT domain accounts for 50,000 events. This establishes an approximate concentration ratio of 18:1 between the dominant client (C6) and each IoMT client, indicating a highly skewed data distribution across the federated topology.

Beyond volume, the trace structure reveals substantial differences between domains. The IoMT partitions exhibit a one-to-one correspondence between events and traces (50,000 events and 50,000 traces), indicating feature-centric observational logging. In contrast, IoT partitions aggregate multiple events within each trace. Client C6 distributes 460,000 events across 20,880 traces, averaging approximately 22 events per trace, while Edge-IIoT clients (C1–C4) exceed 400 events per trace in several cases. This difference highlights two distinct behavioral granularities: extended temporal sequences in IoT vs. discrete, patient-centric observations in IoMT.

**Figure 3:**
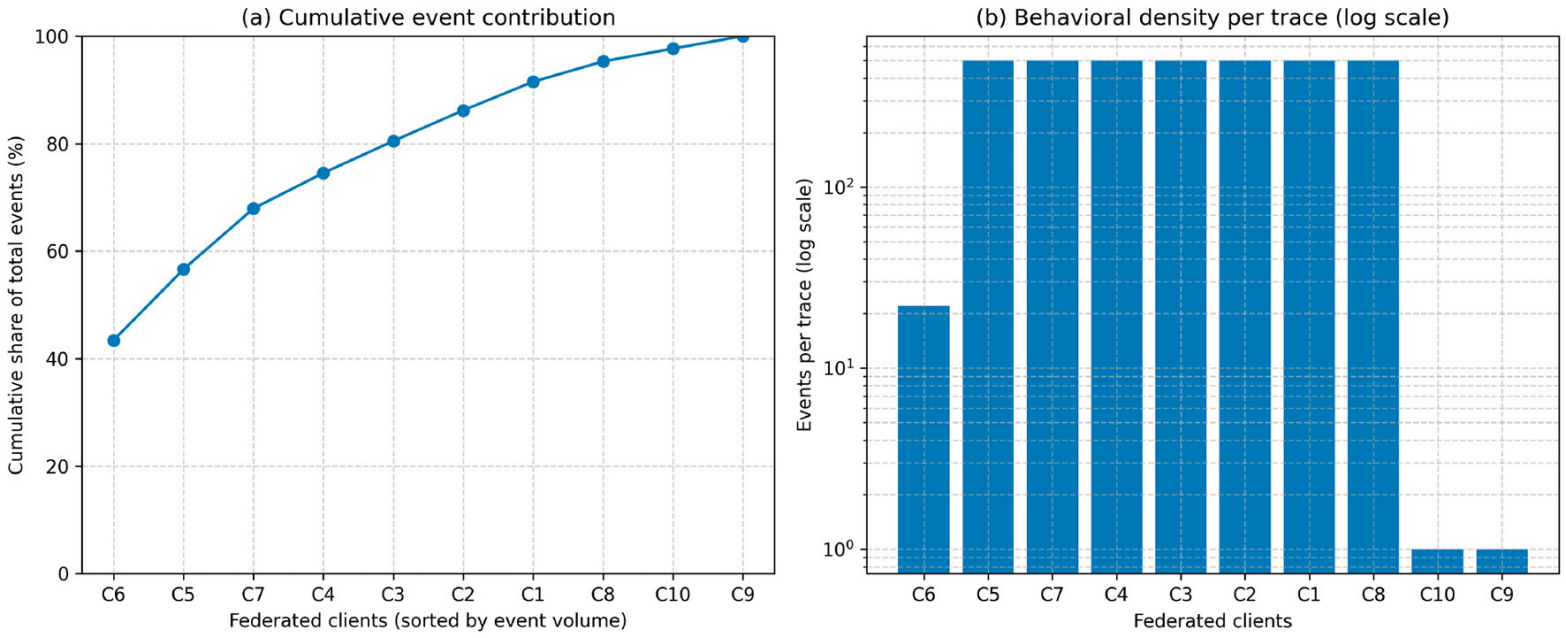
Structural non-IID profile of the experimental setup. **(a)** Cumulative event contribution across federated clients sorted by data volume. **(b)** Behavioral density per trace (log scale).

Figure 3 provides a structural representation of these characteristics. Figure 3a shows the cumulative contribution of events after ordering clients in descending order by volume. The first client contributes approximately 43% of the total, and the cumulative curve exceeds 70% when the three largest clients are included. The curve reaches approximately 90% before incorporating the IoMT partitions, confirming their limited contribution to the overall event mass.

Figure 3b shows the behavioral density per trace on a logarithmic scale. IoT clients are concentrated in the upper region, reflecting high event aggregation per trace, while IoMT clients are located at the lower bound, with values close to 1. The separation spans more than two orders of magnitude, indicating strong structural heterogeneity in trace composition.

In the centralized scenario, full aggregation produces a dataset of 1,060,000 events distributed across 71,980 traces. Under this configuration, volumetric and structural asymmetries are internally averaged, effectively masking inter-client differences. In contrast, the federated scenario preserves both the concentration of data mass and the heterogeneity of trace structures across clients.

These characteristics define a highly non-IID environment with two independent sources of heterogeneity: (i) volumetric imbalance, driven by the concentration of events in a small subset of clients, and (ii) structural heterogeneity, reflected in the large differences in event density per trace between IoT and IoMT domains. This combination creates a challenging setting for FL, as it introduces heterogeneous probability distributions across domains under federated aggregation.

### 4.2 Anomaly detection performance: centralized vs. federated

A comparative evaluation of the centralized regime and the FedAvg federated scheme was performed on the previously defined IHD, using the same training and test partitioning at the trace level, the same standardization protocol, and a fixed decision threshold of 0.5. Table 2 presents the aggregated global performance metrics.

**Table 2:** Overall performance in trace-level anomaly detection: comparison between centralized and federated training.

Model	Precision	Recall	F1	Balanced accuracy	AUC-ROC
Centralized-IHD	0.969	0.993	0.981	0.963	0.995
Federated-FedAvg	0.998	0.530	0.692	0.764	0.995

The results reveal a clear divergence between the two schemes. Centralized training achieves an F1 score of 0.981, with a recall of 0.993 and a precision of 0.969, indicating near-complete detection of anomalous traces while maintaining a controlled false-positive rate. The balanced accuracy (0.963) confirms stable performance under class imbalance.

A difference, the federated model maintains the same AUC-ROC (0.995 vs. 0.995), indicating that the discriminative ranking of instances is preserved under decentralization. However, recall drops to 0.530, reducing F1 to 0.692 and balanced accuracy to 0.764. This behavior reflects a misalignment between the predicted probability distribution and the fixed decision threshold under the federated regime.

The coexistence of a high AUC-ROC with a substantial reduction in recall indicates that the model retains its ability to rank traces by anomaly likelihood but fails to activate positive classifications at the same rate. This behavior is further detailed in Table 3, which presents the decomposition of the confusion matrix.

**Table 3:** Trace-level error decomposition: comparison between centralized and federated training.

Model	TN	FP	FN	TP
Centralized-IHD	6,794	484	101	15,319
Federated-FedAvg	7,264	14	7,246	8,174

TN, true negatives; FP, false positives; FN, false negatives; TP, true positives.

The centralized model produces 101 false negatives and 484 false positives, maintaining a balance between both error types. In contrast, the federated model reduces false positives from 484 to 14, while increasing false negatives from 101 to 7,246. This shift indicates a systematic change in classification behavior, where the model adopts a conservative regime that strongly favors negative predictions.

**Figure 4:**
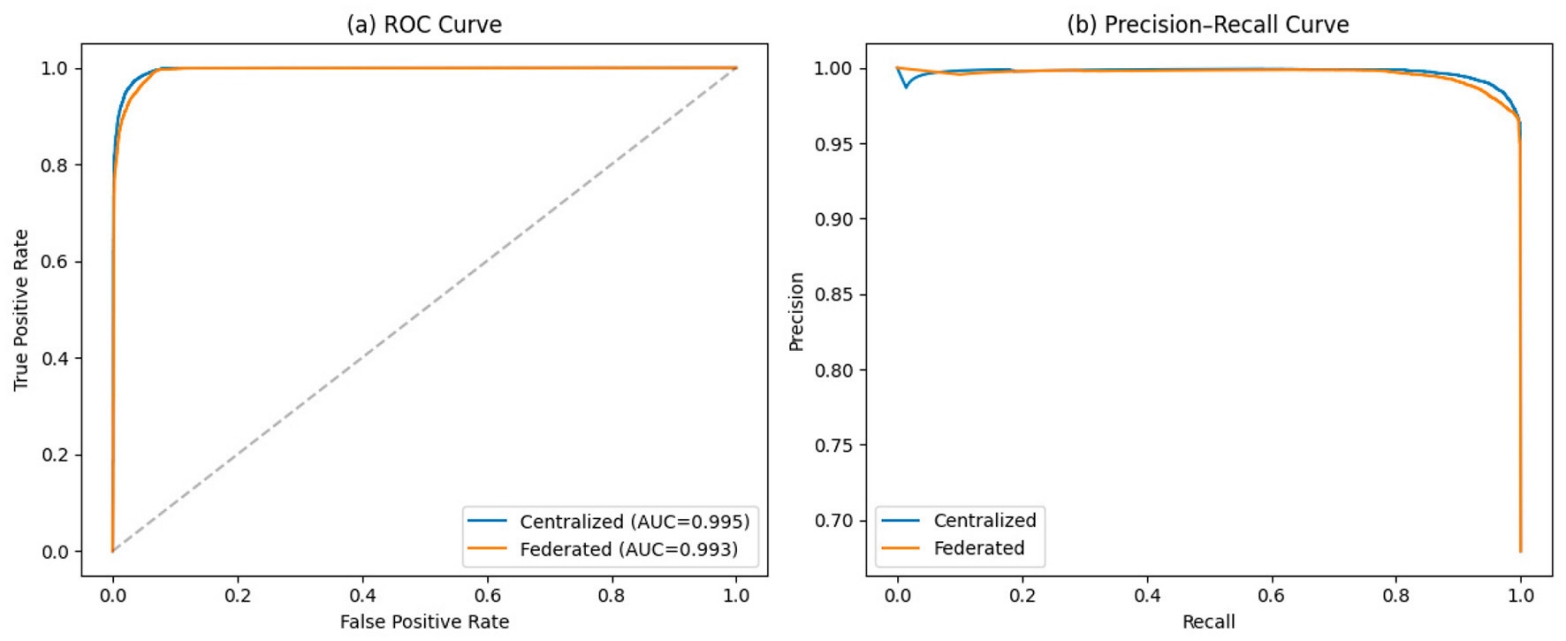
Comparison of discriminative behavior between centralized and federated training: **(a)** ROC curve; **(b)** Precision-Recall curve.

Figure 4 further illustrates this behavior. Figure 4a shows that the ROC curves of the centralized and federated models overlap almost completely across the entire range of false positive rates, confirming that both models exhibit equivalent ranking performance.

However, Figure 4b shows a divergence in the precision-recall relationship. While both models maintain high precision across a wide range of thresholds, the federated model exhibits a steeper decline in recall as the threshold increases. This indicates that the distribution of predicted scores is more concentrated below the decision threshold, reducing sensitivity despite preserved separability.

### 4.3 Impact of non-IID data on federated behavioral inference

Disaggregated evaluation at the client level enables a direct examination of how non-IID heterogeneity affects model sensitivity under federated aggregation. Table 4 presents stratified performance by client (C1–C10) and by domain (IoT, IoMT), including test set size (*n*_test_), anomaly proportion (π_anomaly_), F1, and Recall for both training schemes, together with their differences (Δ).

**Table 4:** Impact of federated training under non-IID heterogeneity at client and domain level.

A	B	C	D	E	F	G	H	I	J
C1	Client	3,049	0.476	0.993	0.957	−0.036	0.997	0.920	−0.076
C10	Client	5,042	0.951	0.975	0.452	−0.522	0.995	0.292	−0.703
C2	Client	2,961	0.460	0.991	0.947	−0.044	0.990	0.900	−0.090
C3	Client	3,037	0.482	0.993	0.964	−0.030	0.996	0.933	−0.063
C4	Client	3,048	0.472	0.992	0.955	−0.037	0.994	0.915	−0.078
C5	Client	192	0.000	0.000	0.000	0.000	0.000	0.000	0.000
C6	Client	192	0.833	1.000	1.000	0.000	1.000	1.000	0.000
C7	Client	192	0.089	0.385	0.417	0.032	0.294	0.294	0.000
C8	Client	26	0.462	0.667	0.400	−0.267	0.583	0.250	−0.333
C9	Client	4,959	0.952	0.976	0.447	−0.529	0.994	0.288	−0.706
IoMT	Domain	10,001	0.951	0.975	0.450	−0.526	0.995	0.290	−0.704
IoT	Domain	12,697	0.465	0.991	0.955	−0.036	0.992	0.917	−0.075

A, Entity; B, Level; C, *n*_test_; D, π_anomaly_; E, F1_Cent; F, F1_Fed; G, ΔF1; H, Recall_Cent; I, Recall_Fed; J, ΔRecall.

Test set sizes reflect the stratified client-level partitioning applied during evaluation. The results show a clear differentiation across clients depending on class distribution. In clients C1–C4, associated with the IoT domain and with anomaly ratios in the range 0.47–0.48, the reduction in recall under federated training remains limited (ΔRecall between −0.063 and −0.090), with corresponding F1 decreases between −0.030 and −0.044, indicating consistent behavior across partitions.

In contrast, clients C9 and C10, both characterized by π_anomaly_≈0.95, exhibit substantial reductions in recall (ΔRecall ≈−0.706 and −0.703) and F1 (≈−0.52). Given their large test sets (*n*_test_>4900), these clients exhibit the largest observed performance shifts in the federated model. The aggregated IoMT domain reproduces this pattern (ΔRecall = −0.704; ΔF1 = −0.526), whereas the IoT domain maintains relatively small reductions (ΔRecall = −0.075; ΔF1 = −0.036).

The relationship between π_anomaly_ and ΔRecall indicates that clients with extreme class distributions are associated with the largest performance shifts. In these cases, the federated model substantially reduces the anomaly detection rate while maintaining discriminative capacity, as previously observed in the stability of AUC-ROC values. This indicates that the observed degradation arises from a misalignment between predicted probability distributions and the fixed decision threshold, rather than from a loss of separability in the learned representations.

**Figure 5:**
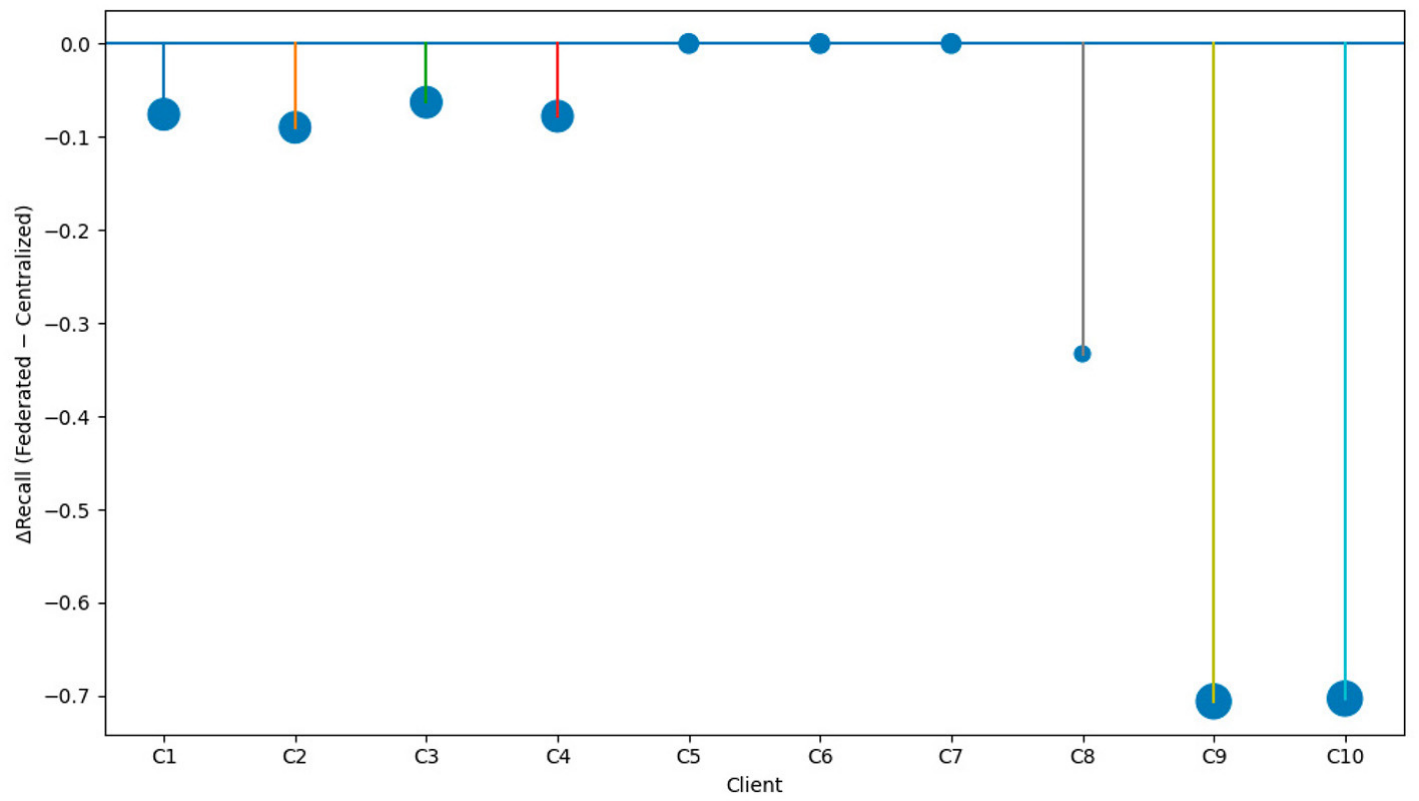
Client-level recall displacement induced by non-IID federated aggregation.

Figure 5 illustrates the displacement in ΔRecall across clients, with marker size proportional to *n*_test_. Clients C1–C4 form a compact cluster with moderate reductions, while C9 and C10 exhibit markedly larger shifts. The relative size of these markers highlights the contribution of IoMT clients to the global behavior of the federated model.

Client C8 exhibits an intermediate reduction in recall (ΔRecall = −0.333), although with limited support (*n*_test_ = 26). Client C7 maintains stable recall (ΔRecall = 0.000) and shows a slight improvement in F1 (+0.032). Client C6 remains invariant, while C5 contains no positive instances in the test set (π_anomaly_ = 0), making anomaly-related metrics undefined for that partition.

### 4.4 Federated learning dynamics and model convergence

Table 5 presents the evolution of the federated model over 20 communication rounds, including global and domain-specific metrics, as well as the magnitude of the parametric shift (Δ*W*_*L*2_).

**Table 5:** Federated learning dynamics and global model stability per round (FedAvg).

A	B	C	D	E	F	G	H
1	0.979	0.991	0.985	0.977	0.975	1.000	84.336
2	0.980	0.992	0.988	0.980	0.975	1.000	50.848
3	0.980	0.993	0.988	0.981	0.975	1.000	35.975
4	0.980	0.993	0.987	0.982	0.975	1.000	51.594
5	0.972	0.978	0.968	0.942	0.975	1.000	48.586
6	0.872	0.775	0.988	0.984	0.784	0.645	32.369
7	0.966	0.963	0.949	0.905	0.975	1.000	52.078
8	0.961	0.956	0.937	0.885	0.975	1.000	52.448
9	0.944	0.905	0.913	0.843	0.962	0.944	38.888
10	0.976	0.977	0.976	0.956	0.976	0.990	58.081
11	0.782	0.643	0.913	0.845	0.682	0.518	56.351
12	0.974	0.980	0.973	0.949	0.975	0.999	42.238
13	0.755	0.608	0.959	0.928	0.580	0.409	57.855
14	0.730	0.576	0.968	0.943	0.516	0.348	56.723
15	0.849	0.738	0.913	0.842	0.805	0.673	38.591
16	0.952	0.915	0.964	0.934	0.944	0.904	53.951
17	0.821	0.698	0.984	0.974	0.690	0.527	52.788
18	0.908	0.836	0.951	0.913	0.880	0.787	41.566
19	0.542	0.373	0.981	0.968	0.007	0.003	36.575
20	0.692	0.530	0.955	0.917	0.450	0.290	54.458

A, Round; B, F1_global; C, Recall_global; D, F1_IoT; E, Recall_IoT; F, F1_IoMT; G, Recall_IoMT; H, Δ*W*_*L*2_.

During the first four rounds, the model operates within a stable performance regime, with *F*1_global_∈[0.978, 0.980] and Recall_global_∈[0.991, 0.993]. In this interval, both IoT and IoMT domains exhibit values above 0.97, indicating consistent behavior across domains in early training stages.

In round 6, a noticeable performance shift is observed, with *F*1_global_ = 0.872 and Recall_global_ = 0.775. Domain-level decomposition shows that this reduction is concentrated in the IoMT subset (*F*1_IoMT_ = 0.784), while IoT maintains values close to 0.99. Between rounds 7 and 18, the learning process exhibits non-monotonic behavior. Partial recoveries are observed (e.g., rounds 10 and 12 with *F*1_global_>0.97), followed by subsequent performance drops (rounds 11, 13, and 14). Throughout this interval, IoT performance remains relatively stable, generally above 0.90, whereas IoMT shows pronounced variability, reaching *F*1_IoMT_ = 0.516 in round 14.

Round 19 corresponds to the most extreme performance degradation, with *F*1_global_ = 0.542 and Recall_global_ = 0.373. In this round, IoT maintains *F*1_IoT_ = 0.981, while IoMT drops to *F*1_IoMT_ = 0.007, indicating a near-total loss of effective classification under the fixed decision threshold in this domain.

In round 20, corresponding to the final model used for evaluation, overall performance stabilizes at *F*1_global_ = 0.692 and Recall_global_ = 0.530. IoT maintains high performance (*F*1_IoT_ = 0.955), whereas IoMT remains degraded (*F*1_IoMT_ = 0.450). The parametric shift Δ*W*_*L*2_ oscillates between 32 and 84 across rounds, without a sustained decreasing trend, indicating persistent variability in model updates across communication rounds. This behavior is consistent with heterogeneous client updates under non-IID conditions, where local gradients introduce conflicting optimization directions across rounds.

**Figure 6:**
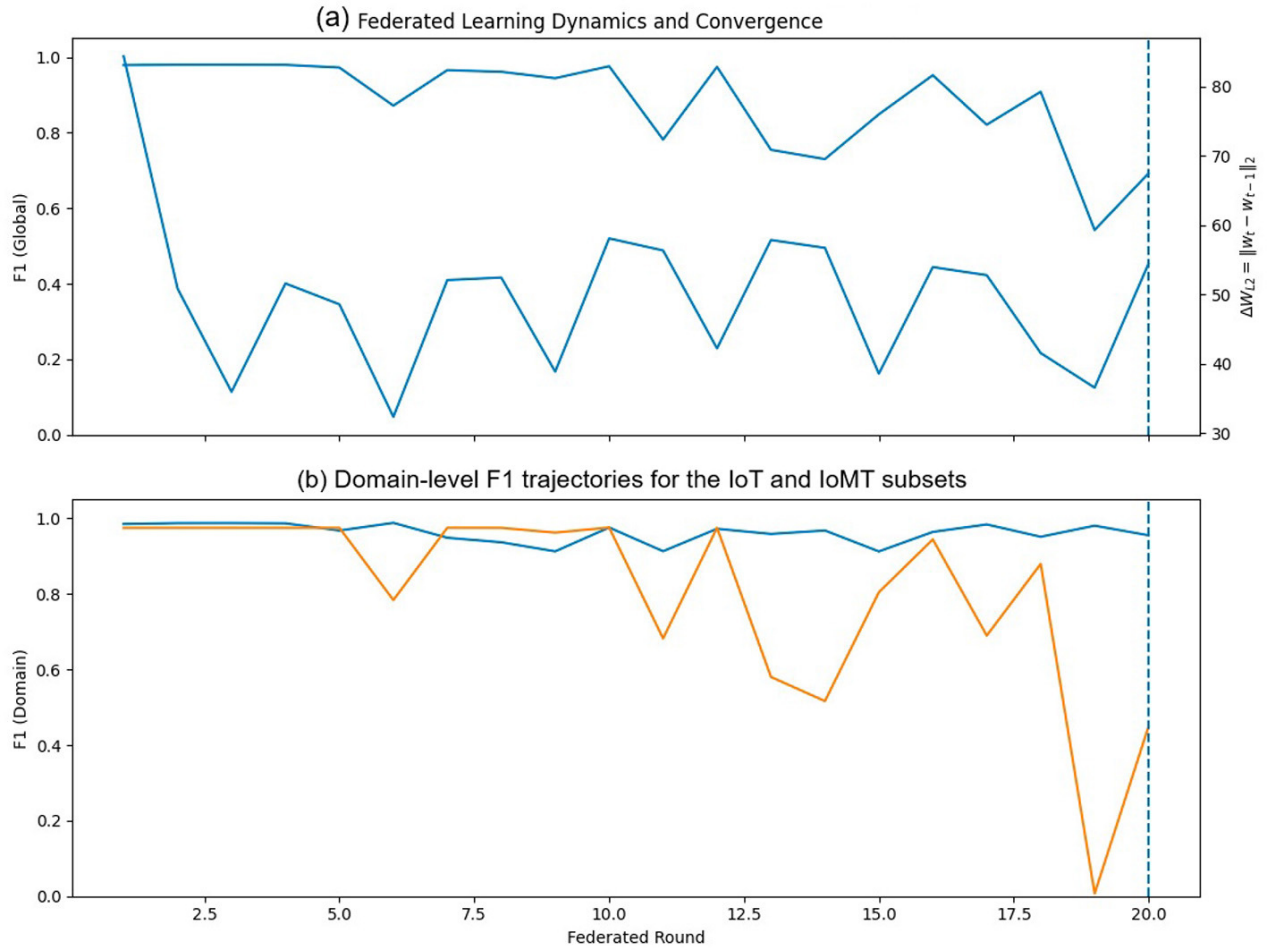
Federated learning dynamics and convergence behavior under FedAvg. **(a)** Evolution of global F1-score and parametric shift Δ*W*_*L*2_. **(b)** Domain-level F1 trajectories for the IoT and IoMT subsets.

Figure 6a shows the evolution of global F1 together with Δ*W*_*L*2_. The global F1 exhibits recurrent oscillations, characterized by abrupt declines followed by partial recovery. The magnitude of Δ*W*_*L*2_ remains variable throughout training, indicating that parameter updates continue to produce substantial changes even in later rounds.

Figure 6b presents the performance trajectories by domain. The IoT curve remains relatively stable across rounds, while the IoMT curve exhibits marked fluctuations, including pronounced drops in rounds 13, 14, and 19.

### 4.5 Structural calibration and probabilistic displacement under federated learning

Table 6 presents the structural evaluation of the model's probabilistic calibration under centralized and federated training, considering both the overall dataset and its domain-wise disaggregation. The reported metrics correspond to the Brier Score, which quantifies the mean squared error between the predicted probability and the actual label, and the ECE with 10 partitions, which measures the discrepancy between the average confidence and the observed empirical frequency.

**Table 6:** Structural calibration evaluation under centralized and federated training.

Entity	*n*_test_	π_anomaly_	Brier score	ECE, 10 bins
Centralized (Global)	22,698	0.679	0.019	0.008
Federated (Global)	22,698	0.679	0.268	0.312
Centralized (IoT)	12,697	0.465	0.006	0.004
Federated (IoT)	12,697	0.465	0.030	0.039
Centralized (IoMT)	10,001	0.951	0.035	0.014
Federated (IoMT)	10,001	0.951	0.571	0.657

Expected Calibration Error (ECE, 10 bins).

Globally, the centralized model exhibits a Brier Score of 0.019 and an ECE of 0.008, values consistent with well-calibrated probabilistic predictions. Under federated training, these indicators increase to 0.268 and 0.312, indicating a degradation in probabilistic calibration.

Analysis by domain reveals that the phenomenon is not uniform. In the IoT domain, the Brier Score increases from 0.006 to 0.030, and the ECE from 0.004 to 0.039. Although an increase is observed, the values remain within a relatively low range, indicating that the probabilistic structure of the federated model remains reasonably aligned with the actual distribution of anomalies in this domain.

In contrast, the IoMT domain exhibits a markedly different behavior. The Brier Score increases from 0.035 to 0.571, and the ECE from 0.014 to 0.657. Given that the proportion of anomalies in this domain is extremely high (π_anomaly_ = 0.951), these values indicate that a considerable fraction of anomalous instances receive low or intermediate predicted probabilities, resulting in a large proportion of anomalies falling below the fixed decision threshold.

The magnitude of the parametric shift between the two models provides additional context for this behavior. The distance between the parameter vectors reaches a value of 69.71 in the Euclidean norm, accompanied by a shift in the intercept of 0.813. These values are consistent with a shift in the effective decision boundary induced by the redistribution of predicted probabilities.

**Figure 7:**
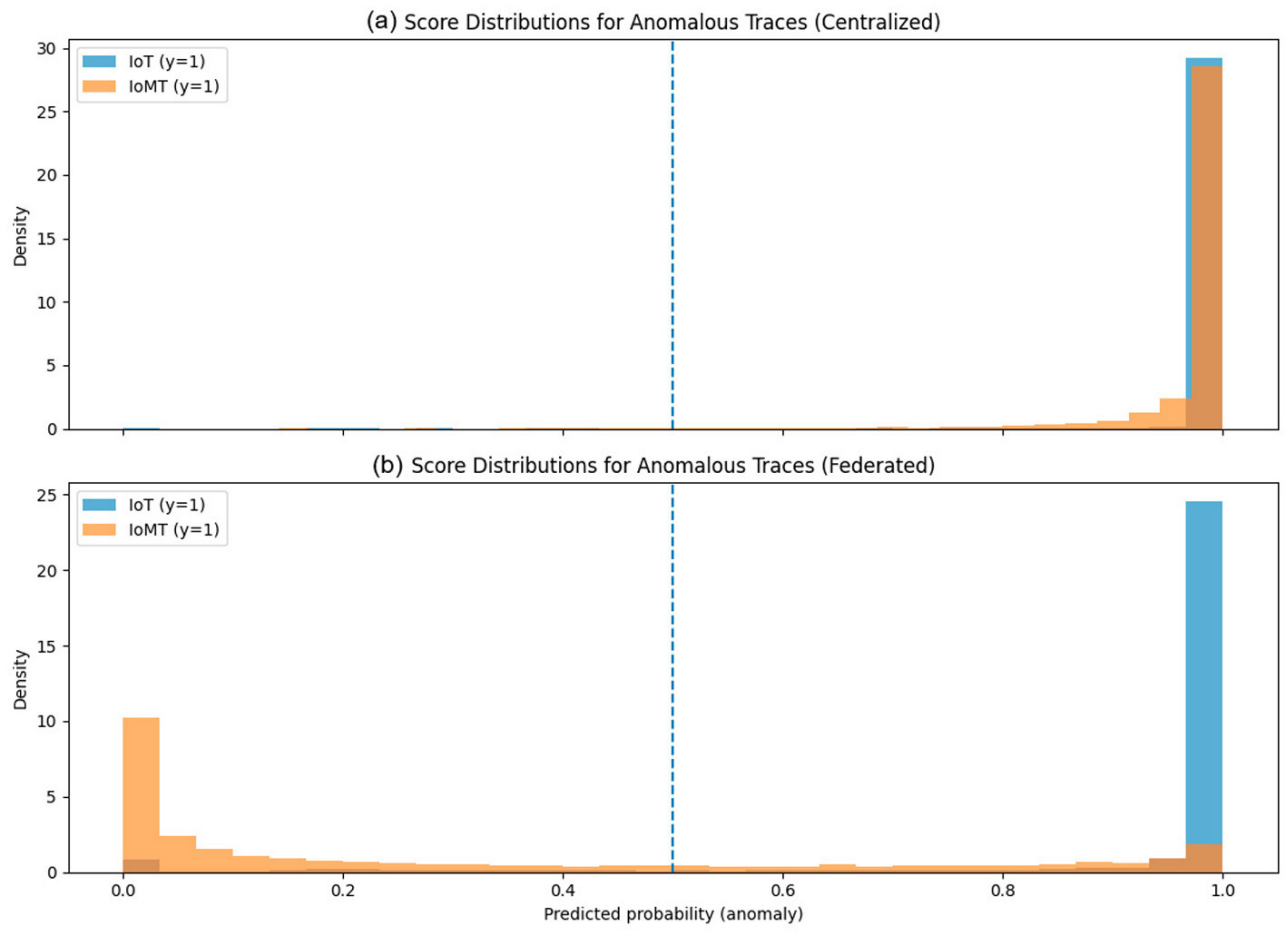
Predicted probability distribution for anomalous traces (*y* = 1) under centralized and federated training: **(a)** Centralized; **(b)** Federated.

Figure 7a shows the predicted probability distribution for anomalous traces (*y* = 1) under centralized training. Both IoT and IoMT exhibit a dominant concentration near 1.0, with the probability mass located above the decision threshold.

In Figure 7b, corresponding to the federated model, the IoT distribution remains concentrated above the threshold with moderate dispersion. In contrast, the IoMT distribution shifts toward lower probability values, with a substantial density below the decision threshold. This redistribution is consistent with the increase in Brier Score and ECE observed for the IoMT domain and with the observed reduction in recall under the fixed decision threshold.

### 4.6 Feature-level attribution under non-IID federation

To further examine whether the probabilistic displacement observed under federated aggregation was associated with changes in the model's internal contribution structure, a SHAP-based feature attribution analysis was performed on the centralized and federated logistic regression models. The analysis focused specifically on anomalous IoMT traces (*y* = 1), corresponding to the domain in which the most severe recall degradation and calibration instability were previously identified.

**Figure 8:**
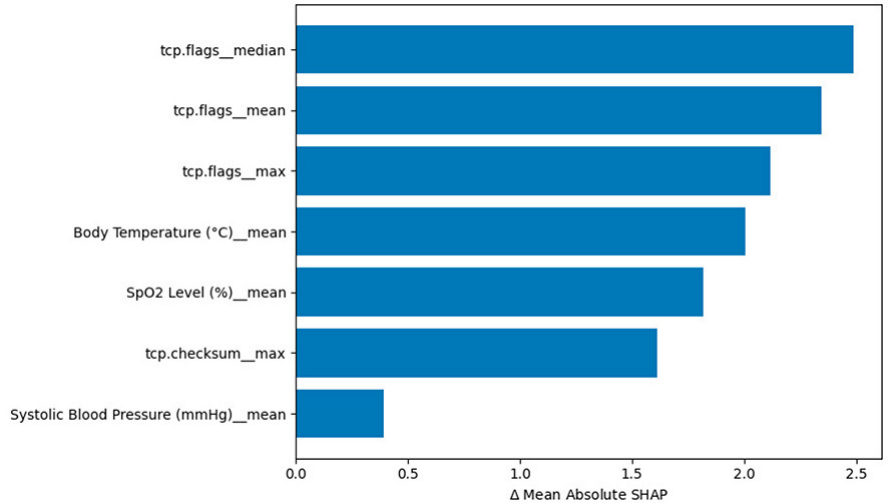
Feature attribution shift under federated aggregation for anomalous IoMT traces. Positive values indicate amplification of feature contribution under federated training.

Figure 8 presents the attribution shift induced by federated aggregation, quantified as the variation in the mean absolute SHAP contribution between the federated and centralized models. Positive values indicate an amplification of feature contribution under federated training.

The attribution structure remains dominated by clinically derived descriptors, particularly oxygen saturation and body temperature, which maintain high contribution magnitudes across both training schemes. However, the federated model exhibits a substantial amplification of network-level descriptors associated with TCP flag statistics. In particular, *tcp.flags__median* and *tcp.flags__mean* exhibit the largest attribution shifts, indicating that federated aggregation redistributes part of the anomaly sensitivity toward lower-level traffic descriptors.

This redistribution is structurally consistent with the probabilistic displacement previously observed. Under centralized training, the dominant contribution structure remains comparatively balanced between physiological and behavioral descriptors, which is consistent with the concentration of anomalous IoMT traces near high-confidence regions of the probability space. Under federated aggregation, the amplification of specific traffic-level descriptors coincides with the broader dispersion of predicted probabilities and with the migration of a substantial proportion of anomalous traces below the fixed decision threshold.

Table 7 further characterizes the structural reorganization of feature importance induced by federated learning. While oxygen saturation variables retain their dominant ranking, TCP flag descriptors experience a substantial increase in relative importance. Conversely, systolic blood pressure descriptors undergo a marked reduction in ranking position, indicating that non-IID aggregation does not uniformly affect all physiological variables.

**Table 7:** Feature attribution ranking redistribution under federated aggregation for anomalous IoMT traces.

Feature	Rank (Centralized)	Rank (Federated)	Rank shift
SpO_2_ Level (%)__mean	1	1	0
Body temperature (°C)__mean	5	6	−1
tcp.flags__median	11	5	+6
tcp.flags__mean	17	11	+6
tcp.flags__max	9	10	−1
tcp.checksum__max	10	12	−2
Systolic blood pressure (mmHg)__mean	12	30	−18

The observed attribution redistribution indicates that the effects of non-IID federation extend beyond calibration degradation alone. The aggregation process alters the relative contribution hierarchy governing anomaly discrimination, amplifying selected traffic-level descriptors while reducing the influence of stability of certain physiological variables. This behavior provides a feature-level interpretation consistent with the severe IoMT recall collapse observed during federated training. It suggests that the degradation arises from a redistribution of discriminative sensitivity rather than a complete loss of anomaly separability.

### 4.7 Post-hoc decision analysis and threshold sensitivity

The evaluation of the federated model under a fixed decision threshold revealed a strong discrepancy between discriminative capacity and operational sensitivity, particularly in the IoMT domain. Despite maintaining a consistently high AUC-ROC across all domains, recall declined significantly under federated aggregation, indicating that the ranking of instances was preserved. At the same time, the decision boundary became misaligned with the underlying data distribution.

At the global level, the federated model, using the standard threshold of 0.5, achieved a precision of 0.998 and an AUC of 0.995, while recall dropped to 0.530. This behavior contrasts with the centralized model, in which recall remained above 0.99 at the same threshold. The imbalance is further reflected in the confusion matrix, where the number of false negatives increased substantially in the federated configuration, despite minimal false positives. This pattern indicates that the model systematically assigns lower probability scores to anomalous instances, causing a large portion of true anomalies to fall below the decision threshold.

This effect is more pronounced in the IoMT domain, where the proportion of anomalies reaches 0.951. Under the fixed threshold, the federated model achieves perfect precision (1.000), but recall collapses to 0.290, with 6753 anomalous instances misclassified as normal. The corresponding Brier score (0.571) and ECE (0.657) confirm a severe misalignment between predicted probabilities and empirical outcomes. In contrast, the IoT domain shows a more stable behavior, with recall remaining above 0.91 and calibration error significantly lower, indicating that the impact of non-IID heterogeneity is not uniformly distributed across domains.

The quantitative impact of this misalignment is explicitly observed in Table 8, where the application of Platt scaling under the same fixed threshold produces a substantial redistribution of predicted probabilities. In the IoMT domain, recall increases from 0.290 to 0.990 while precision remains high (0.961), reducing false negatives from 6753 to 98. This adjustment is accompanied by a drastic reduction in calibration error, with the Brier score decreasing from 0.571 to 0.035 and the ECE from 0.657 to 0.007, indicating a near-complete restoration of probabilistic alignment.

**Table 8:** Post-hoc calibration effects on federated model performance across domains.

Domain	Configuration	Precision	Recall	F1	F1_*macro*_	BA	AUC	Brier	ECE
Global	FedAvg (Fixed 0.5)	0.998	0.530	0.692	0.680	0.764	0.995	0.268	0.312
Global	FedAvg + Platt (Fixed 0.5)	0.949	0.999	0.973	0.955	0.942	0.994	0.029	0.048
IoMT	FedAvg (Fixed 0.5)	1.000	0.290	0.450	0.288	0.645	0.940	0.571	0.657
IoMT	FedAvg + Platt (Fixed 0.5)	0.961	0.990	0.975	0.645	0.607	0.940	0.035	0.007
IoT	FedAvg (Fixed 0.5)	0.997	0.917	0.955	0.960	0.957	0.998	0.030	0.039
IoT	FedAvg + Platt (Fixed 0.5)	0.955	0.995	0.975	0.976	0.977	0.997	0.016	0.041

At the global level, the calibrated model achieves a recall of 0.999 and an F1-score of 0.973, compared to 0.530 and 0.692 in the uncalibrated federated configuration. Similarly, in the IoT domain, recall increases from 0.917 to 0.995, with a consistent reduction in Brier score. Across all domains, the AUC-ROC remains stable, confirming that the calibration process does not alter the relative ordering of instances but rather corrects the scale of predicted probabilities used for decision-making.

The comparison between the uncalibrated and calibrated configurations reveals a consistent shift in the distribution of predicted probabilities across all domains. In the federated setting, anomalous instances in the IoMT domain are predominantly assigned scores below the decision threshold, resulting in many false negatives at the fixed operating point. After calibration, the same instances are redistributed toward higher probability values, increasing their likelihood of surpassing the threshold and being correctly classified. This redistribution is reflected in a reduction in false negatives and a corresponding increase in recall. At the same time, the relative ranking of instances remains stable, as indicated by the AUC-ROC values.

## 5 Discussion

Trace-level results reveal a clear separation between discriminative capacity and decision behavior under federated training. The overall AUC remains virtually unchanged between centralized and federated models, while the federated configuration shifts toward a high-precision regime with a marked reduction in recall. This indicates that the decision function's ranking capability is preserved, whereas the alignment between predicted probabilities and the fixed decision threshold is affected under non-IID aggregation.

The degradation is not uniformly distributed across clients and domains. Table 4 shows that IoT clients with moderate anomaly prevalence exhibit limited reductions in recall and F1, whereas IoMT clients with prevalence close to one experience substantial drops in both metrics. This behavior is consistent with the influence of highly skewed local distributions, where the aggregated model tends to favor configurations that reduce false positives while increasing false negatives in high-prevalence domains.

Federated degradation is therefore concentrated in specific subpopulations rather than being uniformly distributed across the topology. The observed behavior is consistent with the influence of clients exhibiting extreme class imbalance, where local updates may bias the aggregated model toward conservative decision regions. This provides a plausible explanation for why IoT performance remains close to the centralized case, while IoMT concentrates the loss of sensitivity. In operational IoMT environments, this effect is particularly relevant, as reduced false alarms may coexist with missed anomalous traces.

The round-by-round analysis further shows that the federated process does not follow a monotonic convergence trajectory. Instead, the model exhibits alternating patterns of performance variation, particularly in IoMT. While IoT maintains relatively stable performance across rounds, IoMT shows pronounced fluctuations, including transient performance collapses. This behavior is consistent with a learning process in which successive aggregations shift the model between regions of higher sensitivity and more conservative configurations, depending on the balance of local updates.

These dynamics can also be interpreted in light of the absence of proximal regularization in the aggregation process. In standard FedAvg, local updates are aggregated without constraining their divergence from the global model, which may lead to instability when clients exhibit highly heterogeneous distributions. Under such conditions, updates from clients with extreme class imbalance can induce abrupt shifts in the global parameter space, contributing to the transient performance collapses observed in specific rounds.

Proximal methods, such as FedProx, introduce an additional regularization term that penalizes the deviation of local updates from the current global model, effectively constraining the magnitude of client-specific drift during training. In the context of the observed behavior, such a mechanism would likely reduce the oscillatory patterns and mitigate the abrupt transitions between high-sensitivity and conservative regimes by stabilizing the aggregation trajectory. This suggests that the functional collapse observed in certain rounds may be due not only to data heterogeneity but also to the unconstrained aggregation dynamics inherent in FedAvg, highlighting proximal regularization as a relevant direction for improving stability under extreme non-IID conditions.

Calibration analysis provides a more precise explanation of these effects. The centralized model maintains a low Brier Score and ECE values, whereas the federated model exhibits substantial increases in both metrics, particularly in IoMT. As calibration deteriorates, predicted probabilities diverge from empirical frequencies, and the fixed decision threshold becomes increasingly misaligned with the underlying distribution. In high-prevalence domains, this shifts the effective decision boundary so that a significant portion of anomalous instances receive scores below the classification threshold, leading to the observed reduction in recall.

Feature-level attribution analysis further supports this interpretation. The SHAP-based evaluation shows that federated aggregation redistributes the internal contribution hierarchy associated with anomaly discrimination, particularly in the IoMT domain. While clinically derived descriptors such as oxygen saturation and body temperature remain dominant under both training schemes, the federated model substantially amplifies the contribution of network-level descriptors associated with TCP flag statistics. Simultaneously, certain physiological variables exhibit reduced relative importance under federated aggregation. This redistribution is consistent with the probabilistic displacement observed in the score distributions, suggesting that non-IID federation modifies the balance of discriminative sensitivity across behavioral descriptors rather than uniformly degrading the underlying ranking capability.

The observed degradation is therefore not solely due to limitations in federated optimization. Still, it is strongly associated with a misalignment between probabilistic outputs and the decision threshold under non-IID aggregation. The post-hoc calibration results further demonstrate that this effect can be substantially mitigated without modifying the underlying model parameters, indicating that a significant portion of the performance loss is attributable to calibration rather than to a loss of discriminative capacity.

Recent work has explored integrating FL with adaptive aggregation and interpretability mechanisms in IoT and IIoT environments. Approaches incorporating proximal regularization, explainability techniques, or adaptive weighting strategies have been reported to improve robustness under certain configurations (Chowdhury et al., 2026; Alzakari et al., 2025; Alatawi, 2025b). In IoMT settings, privacy constraints and trust requirements further emphasize the need for distributed and auditable learning systems (Jiang et al., 2026; Shammar et al., 2025). Within this landscape, the present results provide empirical evidence that non-IID heterogeneity leads to localized performance degradation, and that this degradation is primarily associated with probabilistic misalignment rather than a loss of discriminative capacity.

The limitations of the study are associated with the chosen experimental configuration. The use of FedAvg without explicit non-IID correction enables direct observation of heterogeneity effects but limits the exploration of alternative aggregation strategies. The use of a fixed decision threshold under conditions of probabilistic misalignment affects the operating point; domain-specific thresholds or Post-hoc calibration can substantially modify recall while preserving AUC. Additionally, the IoMT subset exhibits extreme class prevalence, meaning that small variations in predicted probabilities can produce large changes in recall, potentially affecting direct transferability to other clinical scenarios.

## 6 Conclusions

This study shows that federated anomaly detection in heterogeneous IoT-IoMT environments cannot be fully characterized using aggregated global metrics alone. Using the trace-level IHD, the results indicate that FedAvg preserves global discriminative capacity, as reflected in stable AUC-ROC values, while producing a shift in decision behavior under a fixed threshold. This shift is manifested as a substantial reduction in recall under federated training, particularly in the IoMT domain, despite near-optimal performance in the centralized setting. These findings indicate that non-IID heterogeneity leads to localized degradation rather than uniform performance loss, concentrating sensitivity reductions in domains with extreme class distributions.

The results show that the observed degradation is consistent with interactions among class imbalance, heterogeneous local updates, and aggregation. Under these conditions, the federated model tends toward configurations that preserve ranking while altering probability assignment around the decision threshold, leading to reduced anomaly detection. The increase in Brier Score and ECE further shows that this behavior is associated with a degradation in probabilistic reliability, particularly in high-prevalence domains such as IoMT.

These findings highlight the importance of multilevel evaluation strategies in federated systems that incorporate not only discriminative metrics but also calibration and domain-level analysis. In safety-critical environments such as IoMT, reliance on global indicators can obscure degradation patterns that directly affect operational performance.

Beyond confirming the impact of non-IID heterogeneity, the study demonstrates that the observed degradation in recall is strongly associated with probabilistic misalignment and can be significantly mitigated through calibration-aware strategies. This provides a more precise and operationally relevant characterization of federated model behavior and highlights the importance of integrating calibration into the evaluation and deployment of anomaly detection systems in IoMT environments.

Future work will explore adaptive aggregation mechanisms and domain-aware calibration strategies to mitigate the effects of non-IID heterogeneity. Additional evaluation under dynamic participation scenarios and across diverse IoMT datasets will further support the development of federated models that maintain both sensitivity and stability under realistic conditions. In addition, future research may incorporate human-in-the-loop validation frameworks, in which domain experts interact with model outputs and probabilistic indicators to assess decision quality, confidence alignment, and operational impact in safety-critical environments.

## Data Availability

The datasets analyzed in this study are publicly available from the following sources: Edge-IIoTset: https://www.kaggle.com/datasets/mohamedamineferrag/edgeiiotset-cyber-security-dataset-of-iot-iiot. TON_IoT: https://research.unsw.edu.au/projects/toniot-datasets. Further inquiries can be directed to the corresponding author.
